# Exploring the Benefits of Nutritional and Chemical Characteristics of Touriga Nacional and Arinto Varieties (*Vitis vinifera* L.)

**DOI:** 10.3390/foods13101535

**Published:** 2024-05-15

**Authors:** Paula Pereira, Maria Lídia Palma, Carla Palma, Carlos Borges, Elisabete Maurício, Ana Luísa Fernando, Maria Paula Duarte, Manuela Lageiro, Ana Fernandes, Nuno Mateus, Marisa Nicolai

**Affiliations:** 1 CBIOS—Research Center for Biosciences & Health Technologies, Universidade Lusófona, Campo Grande 376, 1749-024 Lisboa, Portugal; p1204@ulusofona.pt (P.P.); p1814@ulusofona.pt (M.L.P.); elisabete.mauricio@ulusofona.pt (E.M.); 2 Center for Natural Resources and Environment (CERENA), Instituto Superior Técnico (IST), Universidade de Lisboa, Av. Rovisco Pais, 1049-001 Lisboa, Portugal; 3 EPCV—School of Phycology and Life Science, Department of Live Sciences, Universidade Lusófona, Campo Grande 376, 1749-024 Lisboa, Portugal; 4 Instituto Hidrográfico, R. Trinas 49, 1249-093 Lisboa, Portugal; carla.palma@hidrografico.pt (C.P.); carlos.borges@hidrografico.pt (C.B.); 5 Faculty of Engineering-BioRG, Universidade Lusófona, Campo Grande 376, 1749-024 Lisboa, Portugal; 6 MEtRICs, Department of Chemistry, NOVA School of Science and Technology, Universidade NOVA de Lisboa, Campus de Caparica, 2829-516 Caparica, Portugal; ala@fct.unl.pt (A.L.F.); mpcd@fct.unl.pt (M.P.D.); 7 INIAV—Instituto Nacional de Investigação Agrária e Veterinária, 2780-157 Oeiras, Portugal; manuela.lageiro@iniav.pt; 8 GeoBioTec Research Center, NOVA School of Science and Technology, Universidade NOVA de Lisboa, Campus de Caparica, 2829-516 Caparica, Portugal; 9 LAQV/REQUIMTE, Chemistry and Biochemistry Department, Science Faculty, Porto University, 4169-007 Porto, Portugal; ana.fernandes@fc.up.pt (A.F.); nbmateus@fc.up.pt (N.M.)

**Keywords:** polyphenols, antioxidant, fibers, proteins, fatty acids, total sugar, micronutrients, semi-metals, grape pomace, *Vitis vinifera*

## Abstract

Environmental degradation leads to an unsustainable food system. In addition to this issue, the consumption of foods that improve people’s health and well-being is recommended. One of the alternatives is undoubtedly the use of by-products of winemaking, namely in the form of grape pomace flour (GPF). To verify the benefits of using the Touriga Nacional and Arinto (*Vitis vinifera* L.) flour varieties, analytical determinations were made to identify and quantify different components. In terms of nutritional characterization, the Touriga Nacional GPF showed results that indicate better nutritional quality than the Arinto GPF. The Touriga Nacional and Arinto samples had protein contents of 10.13% and 8.38%, polyunsaturated fatty acids of 6.66% and 5.18%, soluble dietary fiber of 14.3% and 1.7%, and insoluble dietary fiber of 55.1% and 46.4%, respectively. The anthocyanins, proanthocyanidins, and flavonols presented in samples were detected by HPLC-DAD/ESI-MS. Atomic absorption spectrometry revealed elevated concentrations of certain elements in Touriga Nacional compared to Arinto, with the former showing higher levels of aluminum (130 mg/kg) and iron (146 mg/kg) against the latter’s Al (120 mg/kg) and Fe (112 mg/kg) content. GPF could become a valuable ingredient due to its nutritional quality and high content of various polyphenols.

## 1. Introduction

One of the main goals expressed in the United Nations Agenda 2030 is to improve the state of nutrition combined with sustainable agriculture to ensure a healthy and quality life for all individuals regardless of their age, gender, or economic power [1]. All of these parameters are related to the fact that there are currently several risk factors associated with diseases that are related to eating habits. With the growing world population—currently 8 billion and estimated be about 10.4 billion in 2080 [2]—it becomes worrying to project the amount of food that will need to be produced to ensure a healthy diet, both in terms of quantity and quality.

According to the Food-Based Dietary Guidelines of the Food and Agriculture Organization of the United Nations (FAO) a sustainable diet goes beyond nutrition and the environment, and is more complex, including economic and socio-cultural dimensions [3]. In this sense, the search for sustainable food production systems that enable the implementation of resilient agricultural practices, with the view of increasing productivity and production, while maintaining a balanced ecosystem; are fit for climate change, and also improve land and soil quality, is becoming inevitable [1].

Grape pomace, composed of skin, seed, and stem, is a disposal from the winemaking industry and has in its constitution bioactive compounds with antimicrobial, anti-inflammatory [4], antioxidant properties [5], anti-cancer effects, and also beneficial cardiovascular and hepatic effects [4,6,7], in addition to other important nutritional components, such as fibers [8], unsaturated fatty acids [9], and proteins [10]; it may also have low total sugars [11] depending on the type of grape, wine, and its production process [10]. The metal composition, including micronutrients, is of high importance in food quality assurance and safety [12].

As previously described in several papers, grape pomace presents in its composition phenolic compounds, such as anthocyanins, catechins, flavonol glycosides, phenolic acids, and alcohols, as well as fibers, namely lignin, cellulose, and hemicellulose; all of these compounds have remarkable effects on health [13].

The use of grape pomace can be a sustainable and very versatile practice that can contribute to human nutrition when used as a food ingredient; it can also be used in compost for plant nutrition. Grape pomace contains various organic and inorganic compounds and essential nutrients such as nitrogen, phosphorus, and macro- and micronutrients that can enhance human health and nutrient cycling [14], contributing to environmental sustainability.

Two distinct cultivars of *Vitis vinifera* L., the white Arinto and the red Touriga Nacional varieties, were subjected to a comparative analysis to elucidate their chemical and nutritional profiles. This study aimed to delineate both the commonalities and divergences in their compositions, thereby providing insights into their unique nutritional and chemical attributes. These findings hold significant promise for innovative applications within the realm of food technology and composting, leveraging the distinct characteristics inherent to each varietal.

## 2. Materials and Methods

### 2.1. Grape Pomace Samples

Pomace samples derived from two distinct *Vitis vinifera* L. varietals, namely Touriga Nacional and Arinto, from red and wine production, respectively, were harvested from the Alentejo region, CARMIM winery, in 2018. Post-collection, these samples underwent a drying process in a J.P. Selecta (Barcelona, Spain) oven, maintained at 60 °C for 24 h. Subsequently, the dried samples were finely milled (approx. 400 µm) using a Moulinex (Alençon, France) domestic blade grinder to achieve a uniform granular consistency and stored in airtight propylene containers at a controlled temperature of −20 °C, until analytical evaluations.

### 2.2. Chemical and Nutritional Composition of Grape Pomace Flour

#### 2.2.1. Nutritional Composition of Grape Pomace Flour

The moisture content of each sample was assessed. This entailed weighing the samples, followed by desiccation in a J.P. Selecta (Barcelona, Spain) oven with forced air circulation set at 105 °C. The process was continued until a constant weight was achieved, adhering to the established procedures delineated in NP 875 [15]. To ensure reproducibility, all assays were conducted in triplicate. Concurrently, the quantification of total ash content in the samples was accurately executed through a process of incineration. This procedure involved the use of a J.P. Selecta-Horn (Barcelona, Spain) muffle furnace, operating at a temperature of 550 °C, in strict accordance with the guidelines stipulated in the Portuguese Norm NP 518 [16].

All the following results were determined based on the dry weight of the sample, so the percentage is always determined about the sample after the drying process.

Utilizing a conversion factor of 6.25, the protein content in GPF samples was ascertained by quantifying total nitrogen, employing the Kjeldahl method. This analysis was conducted by the procedures outlined in ISO 1871:2009 [17], which provides a standardized approach for nitrogen determination in food products. This method is critical for accurately determining the protein content in GPF samples, offering vital insights into their nutritional composition.

Using nuclear magnetic resonance expressed in ISO 8292:2008, the lipid content was quantified using samples previously dried at 50 °C and stabilized [18].

Insoluble dietary fibers (insoluble hemicellulose, cellulose, and lignin) were determined according to Goering and Van Soest [19]. This procedure involves a sequential extraction with neutral and acid detergents, followed by treatment with 72% sulfuric acid and incineration at 550 °C of the final residue. The difference in weight between the neutral and acid detergent extracts represents the insoluble hemicellulose fraction. The acid detergent residue contains lignin, cellulose, and ash. Treatment with 72% sulfuric acid hydrolyzes the cellulose and leaves lignin and ash. The final incineration of the residue in a muffle Heraeus Electronic (Shanghai, Singapore) makes it possible to determine the total content of lignin [19,20]. The soluble dietary fiber percentage was determined by subtracting the sum of the percentage contents of ash, fat, protein, simple sugars [21], and insoluble dietary fiber from the total percentage.

Total carbohydrates were determined gravimetrically, according to the Munson and Walker procedure [22]. This technique is based on the quantification of the cuprous oxide precipitate formed after copper(II) reduction, at high temperatures and alkaline conditions, by the reducing sugars obtained after sugar acid inversion.

#### 2.2.2. Metals and Semi-Metal Composition of Grape Pomace Flour

The determination of the metals aluminum (Al), cadmium (Cd), chromium (Cr), copper (Cu), iron (Fe), lithium (Li), manganese (Mn), nickel (Ni), lead (Pb), and zinc (Zn) and semi-metal arsenic (As) concentrations in the samples was performed by atomic absorption spectrometry in a Solaar-Thermo elemental, Cambridge, United Kingdom, after digestion in a microwave oven ETHOS PLUS Milestone (Sorisole, Italy). The digestion was performed with 10 mL of nitric acid in a three-step procedure: 5 min at 100 °C and 250 watts, 10 min at 180 °C and 800 watts, and 20 min at 180 °C and 800 watts. The procedures were performed according to EPA Method 3051 (2007) [23]. Measurements were performed by validated procedures, and their quality was controlled through the analysis of blank samples; control standards close to the LOQ; and in the middle of the calibration interval, duplicate samples and spiked samples.

Mercury (Hg) concentrations were directly measured in samples by atomic absorption spectrometry with thermal decomposition, using a Direct Mercury Analyzer (DMA Milestone, Sorisole, Italy). Blanks were prepared using the same procedure, without a sample. All reagents were of Merck Suprapure quality, and pure water was Milli-Q grade [12,24].

#### 2.2.3. Polyphenolic Composition of Grape Pomace Flour

The extraction of polyphenolic compounds was performed in triplicate, according to Pérez-Ramírez et al. (2018) [25]. A hydroalcoholic solution and acetone/water mixture were used to extract free phenolics. Then, 0.5 g of freeze-dried grape pomace was mixed with 20 mL water/methanol (50:50, *v*/*v*, pH 2). The mixture was shaken at room temperature for 1 h, and the resulting extract was centrifuged for 15 min at 4 °C and 10,000 rpm (5600× *g*) Dynamica Velocity 14R Refrigerated Centrifuge, Dynamic Scientific Ltd. (Livingston, United Kingdon). The residue was re-extracted with 20 mL of acetone/water (70:30, *v*/*v*), as described above. The supernatants of each extraction were combined, corresponding to the extractable phenolic compounds fraction.

The total polyphenolic content (TPC) on each sample was determined through the Folin–Ciocalteu colorimetric method [26,27]. The reaction mixture was prepared by mixing 15 µL of each extractable phenolic compound fraction (or water for control) with 75 µL of Folin–Ciocalteu phenol reagent and 500 µL of distilled water in 2 mL Eppendorf tubes. The mixture was vortexed for 10 s, followed by the addition of 300 µL of 5% Na_2_CO_3_. The mixture was brought up to 1500 µL by adding 610 µL of distilled water and vortexed for another 10 s. After 30 min of incubation at room temperature in the dark, the absorbance was measured at 750 nm. The results were expressed as mg gallic acid equivalents per g of dry weight (mg GAE/g DW).

Anthocyanins content in the extractable polyphenolic compounds fraction were analyzed using HPLC-DAD [28], on an Elite LaChrom Merck Hitachi HPLC, (Shanghai, China) composed of the following modules: quaternary pump (L-2130), auto-sampler (L-2200), thermostated column compartment (L2300) with a LiChrosphere^®^ 100 LiChroCART^®^ C-18 reverse-phase column (250 mm × 4 mm, i.d.; 5 µm), and thermostated at 25 °C and a diode array detector (L-2455). The mobile phase consisted of HCOOH/H_2_O (10/90, *v*/*v*; solvent A) and HCOOH/CH_3_CN/H_2_O (10/30/60, *v*/*v*/*v*; solvent B). The flow rate was 1.0 mL.min^−1^, with a linear gradient ranging from 80% of A to 15% in 70 min, and then reaching 100% B in 5 min, a final isocratic gradient of 100% B during 10 min, and a final re-equilibration isocratic with 80% A for 5 min. Spectra were recorded between 220 nm and 600 nm, and detection was carried out at 280 nm and 520 nm as the preferred wavelengths. The results were expressed as mg malvidin-3-*O*-glucoside equivalents/g dry weight (mg Mv3Glc/g DW).

To analyze the content of non-anthocyanin compounds, the extractable phenolic compound fractions were evaporated to 1/3 of the original volume under vacuum at 37 °C on a rotary vacuum evaporator, and the volume was adjusted with water to 20.0 mL. The fractions were extracted three times with 20.0 mL ethyl acetate, and the resulting organic phases were combined and evaporated to dryness on a vacuum rotative evaporator at 37 °C. The residues were resuspended on 2.0 mL methanol and analyzed by HPLC-DAD [29]. The mobile phase consisted of HCOOH/H_2_O (1/99, *v*/*v*; solvent A) and CH_3_CN (solvent B). The flow rate was 0.5 mL.min^−1^, with a linear gradient ranging from 90% of A to 65% in 50 min, and then it reached 100% B in 5 min, a final isocratic gradient of 100% B during 7 min, and a final re-equilibration isocratic gradient of 90% A for 5 min. Spectra were recorded between 220 nm and 400 nm, and detection was carried out at 280 nm and 350 nm as preferred wavelength. The results were expressed as mg catechin equivalents/g dry weight (mg catechin/g DW) or as mg quercetin-3-*O*-glucoside equivalents/g dry weight (mg quercetin-3-*O*-glucoside/g DW).

The identification of polyphenolic compounds was performed by HPLC-DAD/ESI-MS, using the same solvents, flow rate, and gradient as for HPLC-DAD analysis (non-anthocyanin compounds). For anthocyanins analysis, solvents were HCOOH/H_2_O (1/99, *v*/*v*) (solvent A) and HCOOH/CH_3_CN/H_2_O (1/30/69, *v*/*v*/*v*) (solvent B), with the same gradient as described for HPLC-DAD analysis. A Finnigan Surveyor series liquid chromatograph equipped with a Thermo Finnigan (Hypersyl Gold) C-18 reversed-phase column (150 mm × 4.6 mm, i.d.; 5 µm, Thermo Scientific) thermostated at 25 °C was used. Detection was carried out between 200 nm and 700 nm, using a Finnigan Surveyor PDA Plus detector. Mass detection was made on a Finnigan LCQ DECA XP MAX Finnigan Cor. (San Jose, CA, USA) quadrupole ion trap equipped with an atmospheric pressure ionization (API) source, using an electrospray ionization (ESI) source. The vaporizer and capillary voltages were 5 kV and 4 V, respectively. The capillary temperature was set at 325 °C. Nitrogen was used for both the sheath and auxiliary gas flow rates of 80 and 30, respectively (in arbitrary units). Spectra were recorded in the negative or positive ion mode between *m*/*z* 120 and 2000. The mass spectrometer was programmed to performed a series of three scans: a full mass, a zoom scan of the most intense ion in the first scan, and an MS-MS of the most intense ion using relative collision energies of 30 V and 60 V.

#### 2.2.4. Nitrogen and Extractable Phosphorus Composition

The determination of nutrient concentrations in the samples (nitrates, NO_x_; ammonia, NH_3_; and extractable phosphorus, PO_4_) was performed by UV/vis spectroscopy, using specific colorimetric methods implemented in a Skalar SANplus Segmented Flow Auto-Analyzer (Breda, Netherlands). NO_x_ and NH_3_ were determined, respectively, according to Strickland and Parsons (1972) [30] and Koroleff (1976) [31], upon KCl extraction, following Bryant and Wright’s method [32]. Extractable phosphorus was determined according to Murphy and Riley (1962) [33], upon Na_2_CO_3_ extraction, following Olsen’s method [34]. All procedures were adapted to segmented flow analysis. The analyses of nutrients in the laboratory were accredited by the Portuguese Accreditation Body, IPAC, following the ISO/IEC 17025:2017 standard for the determination of these parameters in saline water (NP EN ISO/IEC, 17025:2018) [35,36].

### 2.3. Statistical Analysis

Data were expressed as mean ± standard deviation (SD), with experiments being performed at least 3 times for each experimental condition. Unpaired *t*-test was used to determine statistically significant differences (*p* < 0.05). All statistical data were processed using GraphPad Prism version 8.0 for Windows.

## 3. Results and Discussion

### 3.1. Nutritional Composition of Grape Pomace Flour

In the realm of oenological by-products, GPF exhibits contrasting nutritional profiles, as detailed in Table 1. This table provides an analytical breakdown of crucial nutritional constituents that are essential for evaluating the potential application of grape pomace flour in food products and health supplements.

The measurement of ash content is essential for adjusting when calculating the energy content of foods [37]. Consuming mineral-rich foods is associated with benefits such as improved bone health and overall growth [38]. The ash content, indicative of mineral presence, is higher in Touriga Nacional (5.49%) than in Arinto (4.02%). These results are in line with the values reported in previous studies, although they are different varieties [39,40].

The moisture content, a key factor affecting shelf life and microbial stability, is significantly higher in Arinto (8.88%) compared to Touriga Nacional (3.99%). Such a difference influences the necessary storage and preservation conditions for these flours [41] These moisture levels are consistent with those reported in other research studies [42,43]. Products with lower moisture content, like the Touriga Nacional GPF, tend to be more stable and less susceptible to microbial degradation, offering benefits in food formulations [44].

The protein content in Touriga Nacional is higher, at 10.13%, compared to Arinto, which has 8.38%. In other grape varieties, specifically Cabernet Sauvignon and Sauvignon Blanc, the protein levels were similarly close, recorded at 10.8% and 7.2%, respectively [39]. These protein levels are similar to those in wheat flour [45], making grape pomace flour a suitable gluten-free alternative for celiacs, potentially improving nutritional quality.

Regarding lipid profiles, Touriga Nacional exhibits higher concentrations of both monounsaturated (MUFA) and polyunsaturated fatty acids (PUFAs), which are associated with beneficial health effects such as enhanced muscle quality and improved antioxidant status [44]. Concerning total lipid content, the percentages observed for Arinto (8.22%) and Touriga Nacional (10.29%) are consistent with those reported in the literature by Sousa and coworkers [46].

The dietary fiber composition is particularly interesting, with Touriga Nacional showing a significant amount of soluble fiber (14.3%) compared to Arinto (1.7%). Soluble fibers are beneficial for cardiovascular health and can aid in lowering blood cholesterol levels. They also play a role in gut health by influencing the gut microbiota, which is critical for humans [47]. Regarding the total fiber content, Touriga Nacional has a higher percentage at 69.4%, compared to Arinto, which has 48.1%. Both values fall within the typical range observed [40,45,46].

The total sugar concentrations are notably higher in Arinto, at 31.3%, compared to only 4.7% in Touriga Nacional. The sugar content can reach up to 70% of the total composition depending on the vinification methods utilized [48]. For nutritional applications, a reduced sugar content is generally favored to mitigate rapid fermentation in the gastrointestinal tract, which can cause digestive disturbances [49].

These findings suggest that Touriga Nacional GPF might be better suited for enhancing nutritional quality as a food ingredient, which could translate into benefits for quality improvements in food and feed products. Future studies should aim to explore the practical applications of these and other grape pomace flours in various dietary formulations, considering their nutrient profiles and the potential benefits they may confer.

### 3.2. Metals and Semi-Metal Composition of Grape Pomace Flour

These same GPF samples were analyzed for their metal and semi-metal content, and the results are shown in Table 2.

The metal content in GPF from the Arinto and Touriga Nacional varieties, as outlined in Table 2, is significant for its inclusion of both non-essential and essential elements. These elements can influence the potential application of grape pomace in human diets and need to be assessed carefully considering their safety and health implications [12].

Non-essential elements such as aluminum (Al) and nickel (Ni) are present in both varieties of grape pomace, but lithium (Li) was not detected in any of the samples. Aluminum, despite being the most abundant metal in the earth’s crust, is not required by the human body and, at elevated levels, is linked to health concerns, such as neurotoxicity. The grape pomace of the Touriga Nacional variety shows a higher aluminum content, which, while typical for plant materials, should be consumed within safe limits to avoid potential health risks [12]. Nickel, also non-essential, is present in negligible amounts, posing minimal health risks, but their intake should still be monitored due to the potential for toxicity at higher levels.

The presence of heavy metals, including arsenic (As), cadmium (Cd), lead (Pb), and mercury (Hg), in both GPFs calls for careful consideration. Mercury’s undetectable levels are beneficial since it is known for its toxicity. For arsenic, cadmium, and lead, although detectable, their concentrations are relatively low. However, their potential for toxicity, even at low exposure levels, warrants regular monitoring to ensure safety for consumption and use in food.

In contrast, the essential elements, chromium (Cr), copper (Cu), iron (Fe), manganese (Mn), and zinc (Zn), which are crucial trace metals, are present and beneficial for various biological functions. Touriga Nacional GPF, with its higher ash content of 5.49%, as compared to 4.02% in Arinto, suggests a richer mineral profile and may offer more significant dietary benefits. These elements are vital for a range of biological processes, including immune function and metabolic processes. However, it is essential to maintain a balanced intake of these metals to prevent the adverse effects of toxicity [12].

The ash content in grape pomace is an indicator of total mineral content, and the higher ash content in Touriga Nacional might enhance its value as a dietary supplement. Nevertheless, the levels of non-essential and heavy metals must be kept within acceptable safety margins [12].

Incorporating grape pomace into the human diet aligns with sustainable dietary practices and can contribute to the circular economy by utilizing agro-industrial by-products. However, continual testing and adherence to safety standards are crucial for ensuring the benefits of these products. The findings resonate with recent studies, highlighting the potential of grape pomace as a source of bioactive compounds and the need to manage waste products responsibly to minimize environmental impacts [12,50].

With their rich content of trace metals, these findings could be a step towards more sustainable food-production practices if used within the safety standards set by the European Commission and other health regulatory bodies.

### 3.3. Polyphenolic Composition of Grape Pomace Flour

The following results *(*Table 3*)* provide an insightful look into the polyphenolic content of Arinto and Touriga Nacional GPF, offering a perspective on their potential functional uses. The profile of extractable polyphenolics, namely anthocyanins, proanthocyanidins, and flavonols, in these flours signifies a substantial bioactive potential [49,51].

In this way, Arinto, with its higher extractable polyphenolics at 25.9 mg GAE/g DW, suggests a robust antioxidant capacity, which can be fundamental in countering oxidative stress [4]. This polyphenolic content may also be indicative of potential anti-inflammatory properties, given the links established between phenolic compounds and inflammation reduction [52].

The total polyphenol content reported in other studies shows considerable variability [53], often yielding results that are less favorable than those we achieved [48].

Touriga Nacional, while lower in overall extractable polyphenolics, contains anthocyanins at 1.62 mg Mv3Glc/g DW. This level not only reflects the potent antioxidant potential but also aligns with the expected ranges observed in similar studies, emphasizing the consistency and reliability of our analytical approaches [52]. These compounds are crucial and usually reflect antioxidant activities [53].

The difference in proanthocyanidin content across two varieties of grape pomace, Arinto and Touriga Nacional, with concentrations of 2.9 ± 0.1 mg and 0.8 ± 0.1 mg of catechin equivalents per gram of dry weight, respectively, illustrates the variability in their phytochemical composition. Negro et al. [54]. detailed the quantification of proanthocyanidin content across several grape varieties, noting the following concentrations: Negroamaro exhibited 1.7 ± 0.6 mg/g DW, Malvasia di Lecce recorded 1.4 ± 0.2 mg/g DW, Primitivo showed 2.1 ± 0.5 mg/g DW, and C. Sauvignon was found to have 1.2 ± 0.2 mg/g DW. These findings illustrate the variability in proanthocyanidin levels among different grape pomace varieties, suggesting potential varietal influences on the polyphenolic profiles and their associated bioactivities.

The polyphenolic profiles of various grape pomace varieties draw attention to flavonols like quercetin 3-*O*-glucuronide. Measurements from Arinto and Touriga Nacional indicate flavonol concentrations of 0.20 ± 0.02 and 0.18 ± 0.04 mg/g of quercetin-3-*O*-glucuronide equivalents per gram of dry weight, respectively. According to the research by Gil-Sánchez et al. [55], the levels of flavanols including quercetin 3-*O*-glucuronide range from 0.16 to 0.37 mg/g, showcasing a consistent presence across analyzed varieties. These slight variations in flavonol content may play a role in the antioxidant capacity of these grape pomaces, potentially enhancing the nutritional value of food products that incorporate them [56,57].

#### 3.3.1. Anthocyanins Composition of Grape Pomace Flour

**Figure 1:**
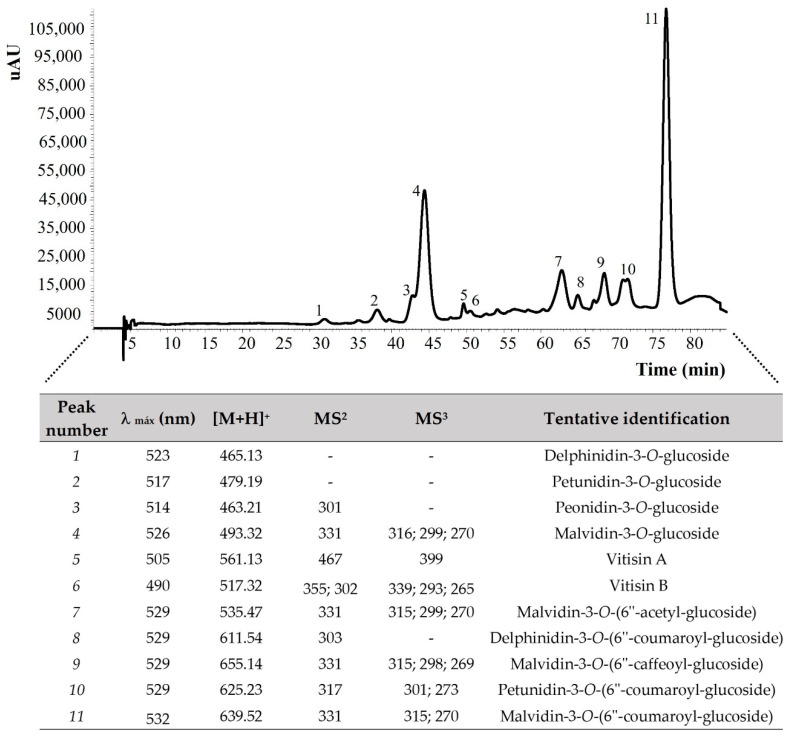
HPLC–DAD/ESI-MS chromatogram and profile of anthocyanins compounds detected in free polyphenol fractions obtained from grape pomace (Touriga Nacional variety). MS^2^—first pseudomolecular ion fragmentation pattern; MS^3^—second pseudomolecular ion fragmentation pattern.

Figure 1 shows the overlay of the chromatogram and table of the anthocyanins of the GPF tentatively identified in fractions with no polyphenols. This analysis provides a deeper insight into the bioactive components of these grape varieties. The results indicate the existence of delphinidin-3-*O*-glucoside, delphinidin-3-*O*-(6″-coumaroyl-glucoside), malvidin-3-*O*-glucoside, malvidin-3-*O*-(6″-acetyl-glucoside), malvidin-3-*O*-(6″-caffeoyl-glucoside), peonidin-3-*O*-glucoside petunidin-3-*O*-(6″-coumaroyl-glucoside), petunidin-3-*O*-glucoside, malvidin-3-*O*-(6″-coumaroyl-glucoside), vitisin A, and vitisin B in the analyzed samples.

Arinto GPF demonstrates an absence of anthocyanins [58], whereas Touriga Nacional is enriched with these potent bioactive compounds. Delphinidin-3-*O*-glucoside, recognized for its potent antioxidant properties, was detected in our samples. This compound is frequently emphasized for its capacity to reduce oxidative stress and inflammation. Consistent with studies by Dimitrovska [58], Yang [59], Moutinho [60], and respective research colleagues, who documented delphinidin-3-*O*-glucoside in various varieties of grape pomace, our findings confirm its presence in GPF samples.

Malvidin-3-*O*-glucoside was another notable discovery in our research. It is especially acclaimed for its cardiovascular advantages and its role in safeguarding against heart disease. This is in line with findings by other researchers [59,60,61,62,63], who have also identified its presence in wine grapes.

The detection of malvidin-3-*O*-(6″-acetyl-glucoside) contributes to the intricacy of the anthocyanin profile in our samples, indicating sophisticated glycosylation. Similar to Dimitrovska and colleagues [58], who identified this derivative in their studies of Cabernet Sauvignon, Merlot, and Pinot Noir grape varieties, our identification highlights the potential for varied anthocyanin modifications in different plant materials.

Both peonidin-3-*O*-glucoside and petunidin-3-*O*-glucoside, recognized for their cardioprotective properties [62], may offer benefits in formulating dietary strategies aimed at enhancing cognitive health. Our results support those of Zhao et al. [63], who noted the consistent presence of these compounds across different *Vitis vinifera* varieties. Future research should focus on examining the varietal influences on their concentration and bioavailability.

The presence of vitisin A and vitisin B, rare stilbenoids, indicates distinctive biosynthetic activity in the sampled grape pomace, associated with notable antimicrobial and anti-inflammatory properties [64]. These compounds are infrequently reported in the literature, with Gonzalez-Arenzana and coworkers [65] observing their occurrence in wine. Their detection in grape pomace suggests specific processing conditions that may encourage their formation.

Further research should aim to explore the quantitative differences in these compounds across various cultivars or geographic regions to assess the environmental or genetic impacts and to examine the bioavailability and metabolic pathways of these compounds in human subjects to better understand their health implications.

#### 3.3.2. Proanthocyanidins and Flavonols Composition of Grape Pomace Flour

**Figure 2:**
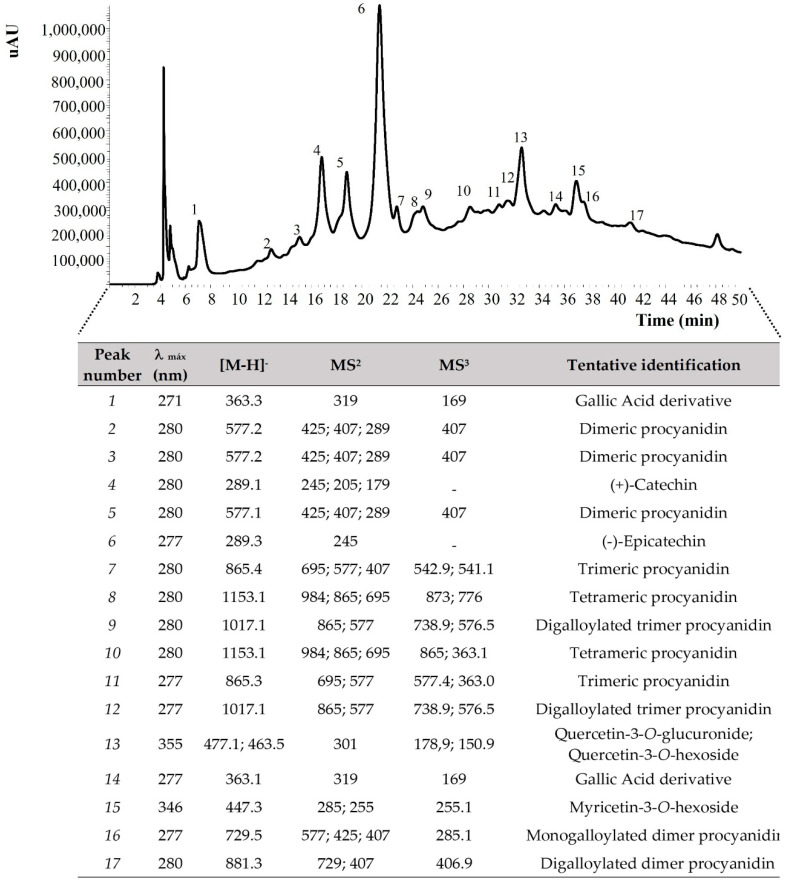
HPLC-DAD/ESI-MS chromatogram and profile of proanthocyanidins and flavonols (non-anthocyanic compounds) detected in free polyphenol fractions obtained from GPF (Arinto variety). MS^2^—first pseudomolecular ion fragmentation pattern; MS^3^—second pseudomolecular ion fragmentation pattern.

The results presented in Figure 2 indicate the presence of several proanthocyanidins, flavonols, and phenolic acids, namely (+)-catechin, (−)-epicatechin, digalloylated dimer procyanidin, digalloylated trimer procyanidin, dimeric procyanidin, gallic acid derivative, monogalloylated dimer procyanidin, myricetin-3-*O*-hexoside, quercetin-3-*O*-glucuronide, quercetin-3-*O*-hexoside, tetrameric procyanidin, and trimeric procyanidin, each with a unique set of potential bioactivities.

The research on grape pomace flour, including a study by Rockenbach et al. (2011), has documented the presence of (+)-catechin and (−)-epicatechin [60,66,67], highlighting their significant contributions to antioxidant capacity. Our findings align with these observations, indicating that these catechins remain stable throughout the pomace processing and preserve their beneficial properties. The uniformity of results across different studies confirms the efficacy of grape pomace flour as a reliable source of these antioxidants.

Our analysis also identified complex procyanidins, such as dimers and trimers, corroborating findings from Ueda et al. [5], who studied grape pomace and seeds from wine production processes. This research suggests that procyanidin levels may differ markedly depending on the grape variety and can be specific winemaking techniques involved.

Moreover, the detection of gallic acid derivatives like monogalloylated dimer procyanidin in our samples mirrors the results from Campos et al. [68], who also investigated their role as antidiabetic compounds. This comparative analysis implies that the processing conditions in our study may enhance the retention or formation of these advantageous derivatives, thereby boosting the benefits of the flour.

Consistent with our observations, Moutinho et al. [60] identified myricetin-3-*O*-hexoside and various quercetin glycosides in grape pomace flour, underscoring their potential role in antioxidant, anti-inflammatory, and antimicrobial activities.

Comparing our results with this study reinforces the notion that grape pomace flour consistently serves as a source of these flavonoids, irrespective of variations in samples and processing methods. This consistency underscores the value of grape pomace flour as a dietary component rich in beneficial phytochemicals.

### 3.4. Nitrogen and Phosphorus Composition

The compositions in nitrate, nitrite, ammonia nitrogen, and phosphorus data for the GPF obtained from wine-making waste are shown below (Table 4).

Regarding compositional values (not accounting for the associated uncertainty), Touriga Nacional exhibits a higher concentration of both forms of nitrogen—nitrate and nitrite—as well as ammonia, compared to Arinto. The presence of nitrate and nitrite levels in grape pomaces is infrequently detailed in the literature. However, it is crucial to examine the results from Arinto (45 mg/kg) and Touriga Nacional (58 mg/kg) GPF. While specific comparative figures are scarce in the literature, our data indicate that the concentration of these components falls below the limits established by the European Union (Regulation No. 1333/2008) [69] for nitrates or nitrites used as additives in food for human consumption. This finding supports a potential discussion on the safe use of this flour as a food ingredient in terms of nitrate and nitrite levels. Additionally, it underscores the role of these compounds in GPF as self-preservatives, suggesting that these flours could be valuable ingredients with high nutritional value that might not require the addition of external preservatives.

The difference in phosphorus content between Arinto (0.83 mg/kg) and Touriga Nacional (101 mg/kg) grape pomace flour is noteworthy. The phosphorus values found for Arinto are lower than those described in the review by Antoníc et al. [49], unlike Touriga Nacional. As for ammonia compounds, although the average values are different, 104 mg/kg for Arinto and 166 mg/kg for Touriga Nacional, there is a probability that the differences are not significant when the estimated uncertainty is considered since the intervals of probable values overlap. Based on these results, it is possible to propose specific and innovative applications for grape pomace flour in the field of composite materials, highlighting its sustainability and the added value it brings in terms of nutrient content, in addition to its rightful application as a food ingredient.

## 4. Conclusions

This research analyzed the chemical and nutritional profiles of two *Vitis vinifera* L. varieties, Arinto and Touriga Nacional, highlighting their distinct attributes and potential applications. Our findings reveal that grape pomace flour (GPF) from these varieties contains beneficial compounds, such as phenolics, unsaturated fatty acids, and fibers, while maintaining a low sugar content, enhancing its value as a sustainable food ingredient.

The marked difference in phosphorus content between Arinto and Touriga Nacional grape pomace flour, along with the analysis of nitrogen compounds, underscores the potential of these flours in various applications, both as nutrient-rich food ingredients and in composite materials. Although the uncertainty intervals for nitrogen values overlap, indicating that the differences might not be statistically significant, the unique compositional traits of each variety emphasize their suitability for specific uses.

Moreover, this study contributes to the goals of the United Nations 2030 Agenda by demonstrating how grape pomace can improve nutrition and promote sustainable agricultural practices. Utilizing GPF supports responsible consumption and production by converting winemaking by-products into valuable nutritional resources, and it also aids in climate action by reducing the carbon footprint associated with waste disposal.

In conclusion, repurposing grape pomace represents a significant step toward innovative food solutions and the sustainable use of resources. It offers a comprehensive approach to achieving key Sustainable Development Goals, providing direct nutritional benefits and broader environmental advantages. This study supports the expanded use and integration of grape pomace in various industries to enhance sustainability and food security.

## Figures and Tables

**Table 1:** Nutritional characterization of grape pomace flour from *Vitis vinifera* L. varieties.

Composition (% *w*/*w* ± SD)	Arinto	Touriga Nacional
Moisture	8.88 ± 0.04 ^b^	3.99 ± 0.14 ^a^
Dry matter		
Ash	4.0 ± 0.03 ^b^	5.49 ± 0.03 ^a^
Proteins	8.38 ± 0.07 ^b^	10.13 ± 0.07 ^a^
Fats/lipids	8.22 ± 0.13 ^b^	10.29 ± 0.07 ^a^
Saturated fatty acids	1.28 ± 0.49 ^b^	0.70 ± 0.35 ^b^
Monounsaturated fatty acids	1.73 ± 0.49 ^b^	2.19 ± 0.26 ^b^
Polyunsaturated fatty acids	5.18 ± 0.72 ^b^	6.66 ± 0.53 ^a^
Dietary fibers		
Soluble fibers	1.7 ± 0.4 ^b^	14.3 ± 0.1 ^a^
Insoluble fibers	46.4 ± 0.6 ^b^	55.1 ± 0.1 ^a^
Hemicellulose	5.7 ± 0.1 ^b^	8.4 ± 0.1 ^a^
Cellulose	15.1 ± 0.4 ^b^	16.7 ± 0.3 ^a^
Lignin	25.6 ± 0.2 ^b^	30.0 ± 0.3 ^a^
Total sugars	31.3 ± 0.4 ^a^	4.7 ± 0.1 ^b^

SD—standard deviation. ^(a,b)^ Within each parameter, values on the same line not sharing lowercase superscript letters indicate statistically significant differences among Arinto and Touriga Nacional (*p* < 0.05). The shaded area corresponds to the percentage values for dry mass.

**Table 2:** Metal content (±uncertainties) in GPF Arinto and Touriga Nacional varieties.

Composition	Arinto	Touriga Nacional
(mg/kg dry sample ± *U*)		
Al	120 ± 24	130 ± 26
As	0.05 ± 0.001	0.10 ± 0.02
Cd	0.12 ± 0.03	0.05 ± 0.01
Cr	0.90 ± 0.15	2.12 ± 0.34
Cu	10.5 ± 1.3	20.8 ± 2.6
Fe	112± 27	146 ± 36
Hg	<0.008	<0.008
Li	n.d.	n.d.
Mn	16.0 ± 1.9	19.8 ± 2.3
Ni	3.36 ± 0.54	0.26 ± 0.04
Pb	0.15 ± 0.02	0.082 ±0.001
Zn	7.27 ± 1.1	8.52 ± 1.2

n.d.—not detected. *U*—expanded uncertainty (for a confidence level of approximately 95%, using a coverage factor of 2).

**Table 3:** Extractable polyphenolics content (determined by colorimetric assay) and individual amounts of specific polyphenolics classes (determined by HPLC-DAD) (±SD) in Arinto and Touriga Nacional GPF varieties.

Composition	Arinto	Touriga Nacional
Extractable polyphenolics (mg GAE/g DW)	25.9 ± 0.3 ^a^	17.1 ± 0.6 ^b^
Anthocyanins (mg Mv3Glc equivalents/g DW)	-	1.62± 0.07
Proanthocyanidins (mg catechin equivalents/g DW)	2.9 ± 0.1 ^a^	0.8± 0.1 ^b^
Flavonols (mg quercetin-3-*O*-Glc equivalents/g DW)	0.20 ± 0.02 ^a^	0.18 ± 0.04 ^a^

SD—standard deviation. In the same line, values with different letters are statistically different.

**Table 4:** Nitrogen and extractable phosphorus from Arinto and Touriga Nacional GPF varieties.

Composition (mg/kg Dry Sample ± *U*)	Arinto	Touriga Nacional
Nitrate and nitrite (mg-NO_3_/kg)	45 ± 11	58 ± 14
Ammonia (mg-NH_3_/kg)	104 ± 26	166 ± 41
Extractable phosphorus (mg-P/kg)	0.83 ± 0.21	101 ± 25

*U*—expanded uncertainty (for a confidence level of approximately 95%, using a coverage factor of 2).

## Data Availability

The original contributions presented in the study are included in the article, further inquiries can be directed to the corresponding author.
